# Palmar Divergent Dislocation of the Scaphoid and Lunate

**DOI:** 10.5334/jbsr.3372

**Published:** 2023-12-29

**Authors:** Wouter Schroven, Luc Walschot, Filip M. Vanhoenacker

**Affiliations:** 1 Department of Orthopedics, University Hospitals, Leuven, Belgium; 2 Department of Orthopedics, AZ Sint-Maarten, Mechelen, Belgium; 3 Department of Radiology, AZ Sint-Maarten and University (Hospital), Antwerp/Ghent, Belgium

**Keywords:** Carpal dislocation, palmar divergent dislocation, scaphoid, lunate

## Abstract

**Teaching Point:** Palmar dislocation of the scaphoid and lunate is an extremely uncommon injury that warrants early diagnosis and treatment to avoid complications such as median nerve dysfunction, avascular necrosis, and premature osteoarthritis.

## Case History

**Figure 1:**
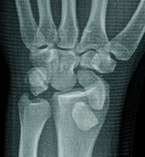
X-ray Frontal view.

**Figure 2:**
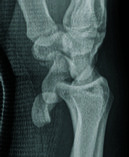
X-ray Sagittal view.

**Figure 3:**
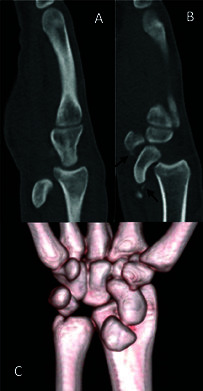
A. Sagittal view of the radiocarpal dislocation B. Sagittal view of the avulsion fractures (arrows) C. Volume-rendered 3D image.

A 22-year-old male presented after a motor vehicle accident, exhibiting left wrist pain, volar swelling, and median nerve paresthesia. Conventional radiography showed dislocation of the lunate into the soft tissues anterior to the radial metaphysis, rotating 250° in the sagittal plane, with the scaphoid’s proximal pole dislocated beyond the radial epiphysis, causing complete anatomical misalignment between the scaphoid and lunate (Figures 1 and 2). A complete disruption of Gilula’s carpal arcs was thus seen on conventional radiography (Figure 1). Subsequent computed tomography (CT) scan confirmed the radiocarpal dislocation (Figure 3A, sagittal reformatted CT image; Figure 3C, volume-rendered 3D image). Additionally, an avulsion fragment between the scaphoid and lunate (Figure 3B, black arrow), volar trapezium (Figure 3B, black arrow), and dorsal triquetrum (not shown) were seen. The avulsion fractures suggested rupture of the volar and dorsal extrinsic wrist ligaments, vital stabilizers of the wrist, and neurovascular conduits of the scaphoid and lunate.

Subsequent surgery revealed complete stripping of the intrinsic and extrinsic ligament attachments from the scaphoid and lunate.

## Comments

Palmar divergent dislocation, an extremely rare occurrence, is defined as the dislocation of the scaphoid and lunate into the carpal tunnel as two separate units due to the complete stripping of ligamentous attachments. It typically follows motor vehicle accidents or falls from height with wrist hyperextension. Common complications associated with this injury include median nerve dysfunction, complex regional pain syndrome, avascular necrosis (AVN), and accelerated osteoarthritis, even after appropriate treatment [1]. The risk of AVN greatly depends on the extent of ligamentous damage, which is suggested by small avulsion fractures. Primary treatment usually consists of early closed reduction and fixation with Kirschner wires. However, since 60% of patients treated this way experience loss of reduction after 6 weeks, open reduction and suturing of the soft tissues are frequently necessary [1]. A primary proximal row carpectomy might be necessary to avoid repetitive operations, particularly if surgery is not performed immediately after presentation [1]. A CT scan is complementary to conventional radiography, since it shows the avulsion fractures, which represent ligamentous injuries that are of neurovascular importance to the carpus. Therefore, a CT scan is essential for diagnosis and guiding surgical treatment.

In conclusion, while immediate open reduction and pinning are the preferred treatments for palmar dislocation of the scaphoid and lunate, radiographic signs may predict the need for primary proximal row carpectomy.
